# Supplementary material for: Oral Intake of Inosine 5′-Monophosphate in Mice Promotes the Absorption of Exogenous Fatty Acids and Their Conversion into Triglycerides though Enhancing the Phosphorylation of Adenosine 5′-Monophosphate-Activated Protein Kinase in the Liver, Leading to Lipohyperplasia
Source: Int J Mol Sci. 2023 Sep 26;24(19):14588. doi: 10.3390/ijms241914588 (PMC10572334; doi:10.3390/ijms241914588)
Supplement: Supplementary file 1 [file ijms-24-14588-s001.zip › ijms-2592595-Supplementary-Materials.pdf]

International Journal of
Molecular Sciences

Supplementary Materials:

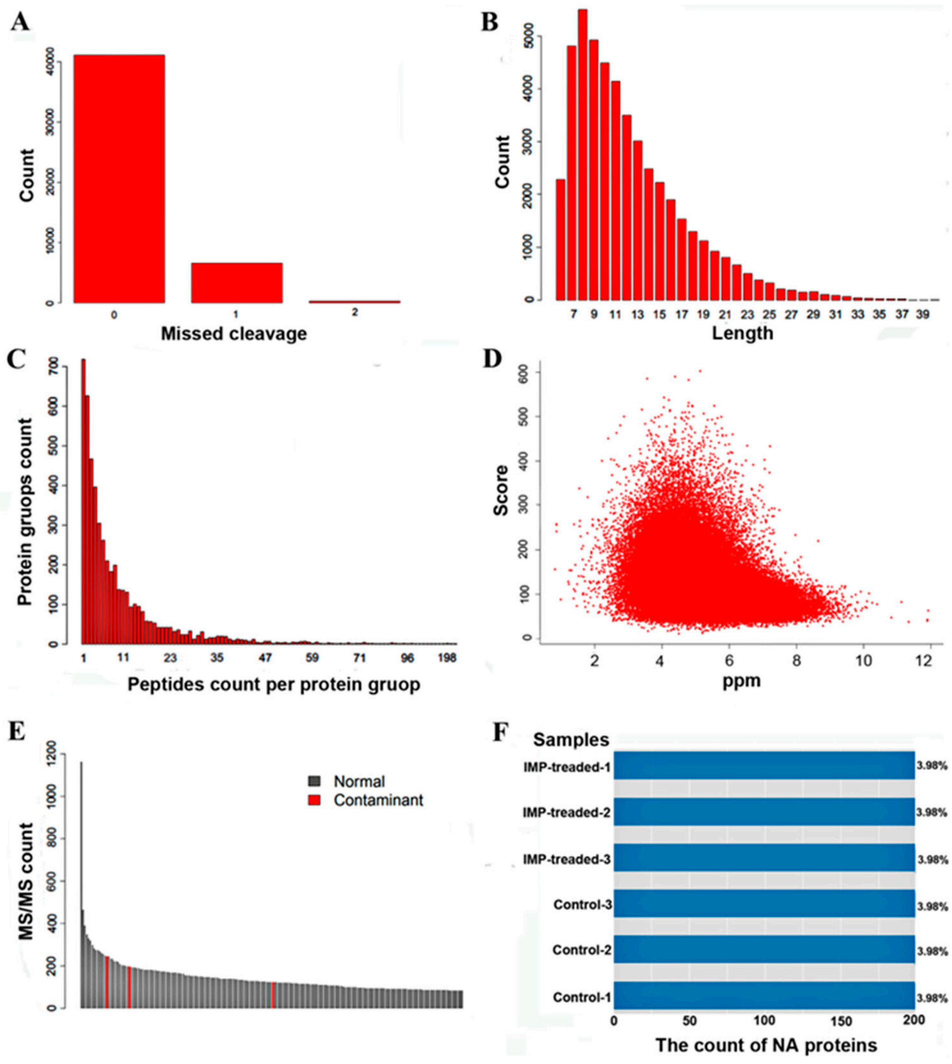

Figure S1. The statistical results used to evaluate of the MS/MS data quality and protein identification. (A) Statistics of the number of target sites that were not lyzed by pancreatic enzyme. (B) Statistics of the length of peptides identified using the MS/MS data. (C) Statistics of the number of the peptides used to identify proteins. (D) Statistics of the mass deviation of all peptide identified based on the MS/MS data (E) Map of the number distribution of contaminated proteins in all identified proteins. (F) The percentage of the proteins lacking quantitative information in total identified proteins.

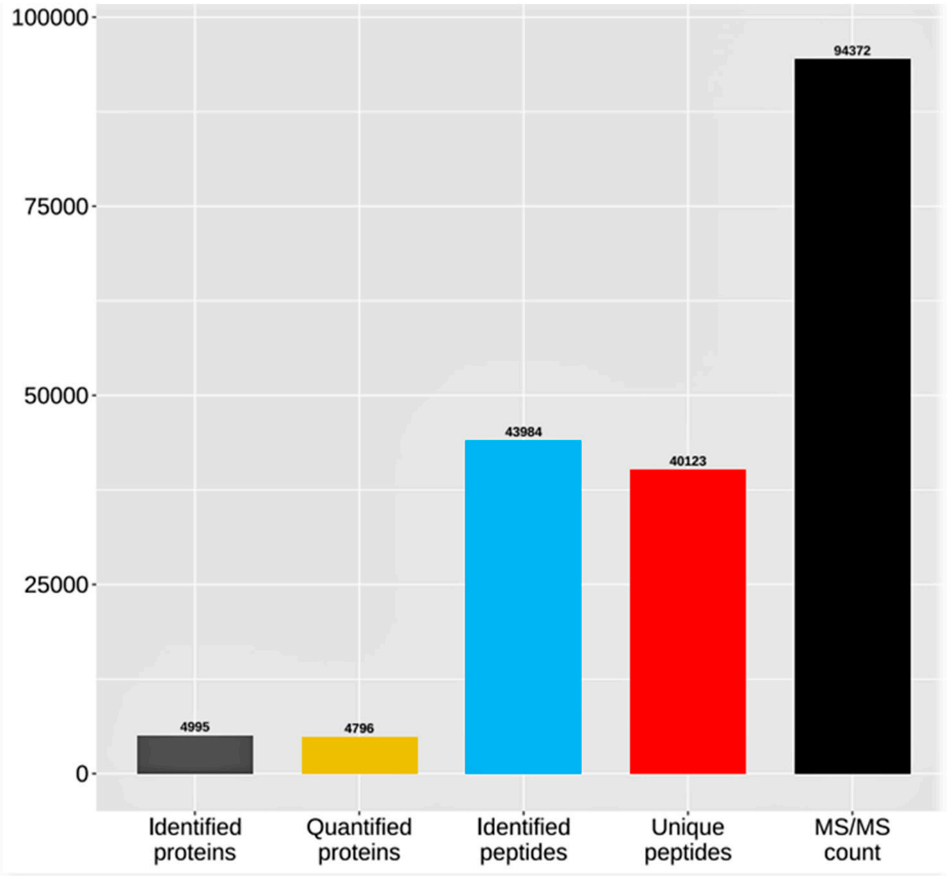

Figure S2. Number of peptides identified based the MS/MS data as well as number of proteins identified based on these peptides.
